# Identification of TRDV-TRAJ V domains in human and mouse T-cell receptor repertoires

**DOI:** 10.3389/fimmu.2023.1286688

**Published:** 2023-11-23

**Authors:** Michael Volkmar, Elham Fakhr, Stefan Zens, Alice Bury, Rienk Offringa, Jessica Gordon, Enes Huduti, Thomas Wölfel, Catherine Wölfel

**Affiliations:** ^1^ TCR Discovery Platform, Helmholtz Institute for Translational Oncology (HI-TRON) Mainz, Mainz, Germany; ^2^ Department D200, German Cancer Research Center (DKFZ), Heidelberg, Germany; ^3^ Helmholtz Institute for Translational Oncology (HI-TRON) Mainz, Mainz, Germany; ^4^ BioNtech, Deptartment Immunotherapies & Preclinical Research, Cellular Biomarker and Immunology Research Team, Mainz, Germany; ^5^ Internal Medicine III, University Cancer Center (UCT), Research Center for Immunotherapy (FZI), University Medical Center (UMC) of the Johannes Gutenberg University Mainz and German Cancer Consortium (DKTK), Partner Site Frankfurt/Mainz, Mainz, Germany

**Keywords:** T cell receptor (TR), TCR, TRDV-TRAJ, hybrid V-domain, V-(D)-J rearrangement, TRDV1

## Abstract

Here, we describe the identification of two T-cell receptors (TRs) containing TRDV genes in their TRA chains, the first one in human and the second one in mouse. First, using 5’RACE on a mixed lymphocyte-tumor cell culture (MLTC), we identified TRDV1 5’-untranslated region (UTR) and complete coding sequence rearranged productively to TRAJ24. Single-cell TR RNA sequencing (RNA-seq) of the MLTC, conducted to identify additional clonotypes, revealed that the analysis software detected the hybrid TRDV-TRAJ TRA (TRA) chain but excluded it from the final results. In a separate project, we performed TR sequencing of tumor-infiltrating lymphocytes (TILs) in a murine tumor model. Here, the predominant clonotype contained a TRA chain with a TRDV2-2-TRAJ49 rearrangement. Again, the hybrid TRA chain was not reported in the final results. Transfection of both TR cDNAs resulted in cell surface localization of TR together with CD3, suggesting a productive protein in both cases. Tumor recognition of the *Homo sapiens* (Homsap) TRDV1-containing TR could be demonstrated by IFN Gamma ELISA ELISpot kit, whereas the *Mus musculus* (Musmus) TR did not recognize a tumor-derived cell line. To determine whether the TRDV-containing TRA chains we detected were rare events or whether TRDV genes are commonly incorporated into TRA chains, we queried the NCBI Sequence Read Archive for TR single-cell RNA-seq data and analyzed 21 human and 23 murine datasets. We found that especially Homsap TRDV1, Musmus TRDV1, and to some extent Musmus TRDV2-2 are more commonly incorporated into TRA chains than several TRAV genes, making those TRDV genes a relevant contribution to TRA diversity. TRDV-containing TRA chains are currently excluded from the final results of V-(D)-J dataset analyses with the CellRanger software. We provide a work-around to avoid exclusion of those hybrid TRA chains from the final analysis results.

## Introduction

The strength of adaptive immunity is its vast antigen receptor diversity that is generated by the rearrangement of D to J and then of V to D-J (for IGH, TRB and TRD chains) and of V to J (for IGK, IGL, TRA, TRG) (1–4). Developing lymphoid cells express the recombinase proteins RAG1 and RAG2 that mediate the recombination of V and D or V and J genes. This recombination event creates the complementarity determining region 3, CDR3, a crucial determinant of antibody or T-cell receptor (TR) specificity. The CDR3-IMGT is limited by two anchors, 2nd-CYS (cysteine C104) and J-PHE or J-TRP (phenylalanine F118 or tryptophan W118) of the J-MOTIF F/WGXG (IMGT label). The CDR3-IMGT with its anchors corresponds to the JUNCTION (3).

Usually, a TRAV-TRAJ gene rearrangement, which encodes a V-alpha domain, is spliced to the TRAC gene to produce a specific TRA chain in CD4^+^CD8^+^ alpha-beta T cells, whereas a TRDV-TRDD-TRDJ gene rearrangement, which encodes a V-delta domain, is spliced to the TRDC gene to produce a specific TRD chain in CD4^-^CD8^-^ gamma-delta T cells. However, there have been identified “promiscuous” V genes that can be part of TRA as well as TR delta chains; the first identified have been designated as TRAV/DV genes. In human, there are five known TRAV/DV genes [*Homo sapiens* (Homsap) TRAV14/DV4, TRAV23/DV6, TRAV29/DV5, TRAV36/DV7, and TRAV38-2/DV8] (2, 5). In mouse, there are 10 *Mus musculus* (Musmus) TRAV/DV genes, eight of them (TRAV4-4/DV10, TRAV6-7/DV9, TRAV13-4/DV7, TRAV14D-3/DV8, TRAV15D-1/DV6D-1, TRAV15-1/DV6-1, TRAV16D/DV11, and TRAV21/DV12) have been found rearranged to TRAJ genes and to TRDD-TRDJ genes (6), whereas the last two, TRAV15-2/DV6-2 and TRAV15D-2/DV6D-2, were assigned to the TRAV15 subgroup on the basis of sequence alignment, although they have not yet been found rearranged to TRAJ genes (6). In contrast, the TRDV genes are generally thought to recombine exclusively with TRDD-TRDJ genes; however, among them, the TRDV1 gene rearranges indeed to TRDD-TRDJ genes and expressed TRD chains as expected, but also to almost (if not all) TRAJ genes and thus expressed hybrid TRDV1-TRAJ-TRAC chains, detected in a sizable population of A13+ (anti-Vdelta1 mAb) TR peripheral lymphocytes (7–10). Using a TR deep sequencing approach on RNA isolated from the peripheral blood mononuclear cells (PBMCs) of three healthy blood donors, Liu et al. (11) indeed identified cDNAs encoding TRDV1-TRAJ TRA chains. Furthermore, TRDV-TRAJ hybrid TRAs have been reported for two TRAs isolated from an HIV patient that recognize HIV peptides presented by human leukocyte antigen (HLA) class I molecules (12, 13).

In this work, we describe two TRs, one of human and one of murine origin, that contain a TRDV gene in their TRA chains. These TRs represent the second most abundant and the most abundant clonotypes in their respective TRA-beta repertoires. Furthermore, to establish that TRDV genes are regularly incorporated into TRA chains, we analyzed multiple publicly available TR repertoire datasets and found TRDV-containing TRA chains in the majority of them.

Note: Throughout this article, we will use the nomenclature of IMGT®, the international ImMunoGeneTics information system®, IMGT (1–6), approved by the HUGO Gene Nomenclature Committee (HGNC) and NCBI Gene, as well as the IMGT unique numbering for TR V and C domains (14, 15). The IMGT IG and TR genes and alleles are written in capital letters (no plural) and are not italicized in publications. The same IMGT rules apply to any other jawed vertebrate species including mouse (for further information, see https://www.imgt.org/IMGTScientificChart/Nomenclature/IMGTnomenclature.html and literature links there).

## Materials and methods

### Tumor cell line

The cell line Ma-Mel-86a used in this study was derived from a 34-year-old female cutaneous nodular melanoma patient (16) and was provided by Annette Paschen (Department of Dermatology, University Hospital, University Duisburg-Essen). For the experiments described herein, previously modified Ma-Mel-86a cells were applied that lacked HLA-class I and -class II expression due to CRISPR/Cas9 knockout of genes B2M and CIITA. In addition, they overexpressed CD80 and CD83 after retroviral transfer to improve their stimulatory capacity. The use of Ma-Mel-86a_KO^B2M/CIITA^_CD80/CD83^hi^ as stimulator of HLA-disparate lymphocytes leads to the enrichment of HLA-independent tumor-reactive T cells.

### T cells

The buffy coat for the healthy donor PBMC isolation was obtained from the blood bank of the University Medical Center of the Johannes-Gutenberg University Mainz, Germany. PBMCs were isolated by Ficoll gradient centrifugation followed by enrichment of CD3^+^ cells by magnetic beads (Miltenyi Biotec GmbH, Bergisch-Gladbach, Germany).

For isolation of murine tumor-infiltrating lymphocytes (TILs) from the pancreatic ductal adenocarcinoma model [described in Gurlevik et al. (17) and Maresch et al. (18)], tumors were resected and dissociated into single cells using the mouse Tumor Dissociation Kit (Miltenyi Biotec). Dead cell staining, unspecific Fc blocking, and antibody staining were performed as described before (19). The following antibodies against mouse were used for extracellular staining: CD45-BV785 (BioLegend, 1:1,000, clone 30-F11, #103149), CD3-FITC (BioLegend, 1:200, clone 17A2, #100204), CD90.2-AF700 (BioLegend, 1:200, clone 20-H12, #105320), CD8a-APC/Cy7 (BioLegend, 1:200, clone 53-6.7, #100714), CD4-BV605 (BioLegend, 1:200, clone RM4-5, #100548), and CD11b-PerCP/Cy5.5 (BioLegend, 1:1,000, clone M1/70, #101228) to sort live CD45+/Cd11b-/CD90.2+/CD3+ T cells and determine their CD8 or CD4 phenotype using a BD FACSAria Fusion cell sorter at the Core Facility Flow Cytometry of the German Cancer Research Center Heidelberg.

### Mixed lymphocyte-tumor cell cultures

Mixed lymphocyte-tumor cell cultures (MLTCs) were generated by coculturing on 96-well plates (in so-called mini-MLTC) per well 1 × 10^4^ CD3^+^ cells isolated from buffy coat lymphocytes of healthy donor BC221 and 5 × 10^3^ irradiated (100 Gy) Ma-Mel-86a_KO^B2M/CIITA^_CD80/CD83^hi^ cells (16). The medium (200 µL/well) was Panserin 401 (PAN-Biotech GmbH, Aidenbach, Germany) supplemented with 10% human serum (Panserin/HS), recombinant human IL-12 (1 ng/µL), and recombinant human IL-7 (5 ng/µL) (both from Miltenyi Biotec). After 1 week, IL-12 was replaced by recombinant human IL-2 (100 units/mL) (Proleukin^®^S, Novartis, Basel, Switzerland), and MLTC responders were stimulated further once per week with irradiated stimulator cells in the presence of IL-2 and IL-7. From day 20 onward, MLTC responders were regularly tested for tumor reactivity using ELISpot (*cf*. **Supplementary Figure S2**).

In parallel to the antigen-driven restimulation of MLTC responders, 1 × 10^5^ tumor-reactive MLTC cells from day 35 were cocultured for 14 days with irradiated (100 Gy) feeder cells (25 × 10^6^ allo-PBMCs derived from a mixture of three buffy coats and 5 × 10^6^ allo-EBV B-lymphocytes), with anti-CD3 antibody (30 ng/mL; clone: OKT3; hybridoma obtained from ATCC, Manassas, VA, USA), with recombinant human IL-2 (250 IU/mL) and with recombinant human IL-15 (2.5 ng/mL). This nonspecific stimulation was used to expand the responder T cells to sufficient numbers for *i.a.* cryopreservation, Beta Mark staining, RNA isolation, and single-cell RNA sequencing (RNA-seq).

### TR 5’RACE

Total RNA was isolated from a tumor-reactive T-cell culture using a Qiagen RNeasy Micro kit (Qiagen GmbH, Hilden, Germany). Here, 100 ng total RNA was used for reverse transcription with the NEB Template Switching Reverse Transcriptase Enzyme Mix (New England Biolabs, Ipswich, MA, USA) according to the recommendations of the manufacturer. We used an oligonucleotide specific for the human TRA chain C region (hsTRAC-RT, see **Supplementary Table S2**) to prime the reverse transcriptase and a template-switching oligonucleotide (TSO) to create a generic primer binding site at the 5’ ends of synthesized cDNAs. The RT product was subjected to two rounds of PCR amplification. All oligonucleotides used were obtained from Merck Sigma-Aldrich GmbH (Darmstadt, Germany). The product of the nested PCR was cloned into pJET (Thermo Fisher, Waltham, MA, USA), and four isolated plasmid clones were subjected to Sanger sequencing (Eurofins Scientific SE, Luxembourg, Luxembourg). Analysis of the sequence data was done using IMGT/V-QUEST (20).

### 10X TR sequencing

The number and viability of isolated T cells were assessed either by manual counting with trypan blue or using a CASY TT cell counter (OMNI Life Science GmbH & Co. KG, Bremen, Germany). VDJ sequencing libraries were established according to the manufacturer’s protocols (CG000331 Revs. D and E with adaptations; 10X Genomics, Pleasanton, CA, USA). Paired-end sequencing was performed either in-house (HI-TRON Mainz, Mainz, Germany) on an Illumina MiSeq sequencer or by the DKFZ Genomics Core Facility (Heidelberg, Germany) on an Illumina NovaSeq6000 sequencer, aiming for ≥5,000 reads/cell. Fastq files were analyzed using the CellRanger vdj pipeline as recommended by the manufacturer (*cf*. Data Analysis). Publicly available V-(D)-J datasets (**Supplementary Table S1**) were downloaded from the NCBI Sequence Read Archive using the SRA Toolkit and analyzed analogously to the in-house data.

### Cloning of TR for *in vitro* transcription and retroviral transduction

We ordered the variable domains (V-(D)-J) of the TR chains as synthetic dsDNA oligonucleotides from Twist Bioscience (San Francisco, CA, USA) and cloned them in-frame with *M. musculus* TRAC and TRBC genes that are stabilized by an additional disulfide bridge (21, 22) using Golden Gate Assembly as described earlier (23) into a pcDNA3.1(+) backbone. The pcDNA3.1 plasmid was purchased from Thermo Fisher (Waltham, MA, USA). After sequence verification via Sanger sequencing, 1 µg of NotI-linearized plasmid was subjected to *in vitro* transcription using the HiScribe T7 ARCA mRNA Kit (with tailing) (New England Biolabs, Ipswich, MA, USA).

For retroviral transduction of primary T cells, we cloned the (V-(D)-J) dsDNA oligonucleotides into pMX-IRES-puro-DEST using Golden Gate Assembly as previously described (24).

### Electroporation of modified Jurkat cells

Jurkat E6-1 cells were obtained from ATCC (Manassas, VA, USA) and grown in RPMI 1640 with 10% FBS and 1% Non-Essential Amino Acids solution (Thermo Fisher). The endogenous TR chains of the Jurkat cells were knocked out using CRISPR/Cas9 targeting the first exon of the TRAC and TRBC genes. For electroporation of TR constructs, 1 × 10^6^ cells were suspended in 20 µL Opti-MEM medium (Thermo Fisher) and 2.5 µg of IVT-RNA was added. For the mock control, the cells were suspended in Opti-MEM without RNA. Cells and RNA were mixed by gentle pipetting, transferred into a Lonza Nucleocuvette^®^, and immediately electroporated using the CL120 program of the 4D-Nucleofector X Unit (Lonza, Walkersville, MD, USA). After electroporation, 80 µL pre-warmed complete RPMI 1640 (Thermo Fisher) medium (37°C) was added and the cells were let to rest for 10 min. Then, the cell suspension was transferred to 1 mL pre-warmed complete RPMI 1640 medium supplemented with 10% FBS in a 48-well plate and incubated at 37°C, 5% CO_2_.

### Transduction of primary T cells

Retroviral particles were produced in Phoenix-Ampho cells that were provided by Matthias Theobald (University Medical Center Mainz, Mainz, Germany) and maintained in DMEM (Thermo Fisher Scientific, Waltham, MA, USA) supplemented with 10% fetal calf serum (FCS; PAN Biotech, Aidenbach, Germany), 1% penicillin/streptomycin, 1% L-glutamine (Sigma-Aldrich, St. Louis, MO, USA), and 25 mM HEPES buffer (Lonza, Basel, Switzerland). In brief, Phoenix-Ampho cells were cotransfected with 5 µg of each of the helper plasmids pCOLT-GALV and pHIT60 and 10 µg of the TR retroviral pMX vector using Fugene 6 (Promega, Madison, WI, USA) according to the manufacturer’s instructions. On the following day, medium was changed to Panserin/HS (*cf*. above) and virus particles were harvested after additional incubation for 16 h.

Parallel to the viral production, CD3^+^ T cells were purified from PBMCs derived from the buffy coat of a healthy donor using the CD3^+^ selection kit (Miltenyi Biotec) and activated for 2 days with an anti-CD3 antibody (30 ng/mL; clone: OKT3) and rhIL-2 (600 U/mL). Subsequently, endogenous TRs were deleted by electroporation of CRISPR/Cas9 RNPs targeting both the Homsap TRAC and TRBC1/2 loci (**Supplementary Figure S1**). CRISPR/Cas9-edited CD3^+^ T cells were spinoculated with virus particles (90 min at 2,000 rpm) in the presence of Polybrene (4 μg/mL; Sigma Aldrich) and rhIL-2 (600 U/mL). One day after transduction, virus supernatant was removed and transgenic T cells were used for the experiments. During coculture, TR transgene selection with puromycin (1 μg/mL; Sigma Aldrich) was maintained. TR expression was confirmed by FACS analysis, and tumor reactivity was tested by IFNγ-ELISpot assays.

### FACS analysis

Forty-eight hours after electroporation, cells were analyzed for TR expression. The cells were first washed using prechilled Cell Staining Buffer (BioLegend, San Diego, CA, USA) supplemented with 2 mM EDTA. Then, the cells were stained with LIVE/DEAD Fixable Green Dead Cell Stain Kit (Thermo Fisher) in a 1:1,000 dilution for 30 min on ice. The samples were washed with Cell Staining Buffer and stained with anti-mouse beta TCR chain Alexa Fluor^®^ 647 and anti-human CD3 PE (both BioLegend) at a concentration of 0.02 µg/µL for 30 min on ice. For primary T cells, anti-human alpha/beta TCR APC (BioLegend), anti-mouse alpha/beta TCR FITC (Origene), anti-IgG-PE, anti-CD8-PE, and IOTest^®^ Beta Mark TCR Vβ Repertoire Kit (Beckman Coulter) were used as recommended by the manufacturers.

Then, the cells were washed and fixed by incubation in Fixation Buffer (BioLegend) for 30 min at room temperature. Subsequently, cells were washed again, passed through a 30-µm filter, and analyzed using a BD FACSAria™ Fusion instrument. Unless indicated otherwise, 10,000 cells were analyzed per condition.

### ELISpot assay

IFNγ-ELISpot assays were performed as described previously (24, 25). Briefly, the endogenous TRs of healthy donor-derived T cells were knocked out as described by us in (24), and the knockout was confirmed by FACS analysis. Tumor cells were seeded (50,000 cells/well) into ELISpot plates (Millipore, Burlington, MA, USA). TR-transfected T cells (10,000 T cells/well) were incubated with or without tumor cells for 20–24 h. Subsequently, the plates were developed and imaged using an ImmunoSpot analyzer device (Cellular Technology Limited, Cleveland, OH, USA).

### Data analysis and visualization

After testing several versions of the CellRanger software, raw 10X V-(D)-J sequencing datasets were analyzed with CellRanger v7.0.1 utilizing the GRCh38-7.1.0 human or the GRCm38-7.0.0 murine reference, respectively. Further analyses and visualizations were performed with R 4.2.0 (26) using the following packages: ggplot2 (27), dplyr (28), and knitr (29, 30).

Flow cytometry results were analyzed with FlowJo™ (v10.8.1, BD Life Sciences), and scatterplots were exported as SVG or PNG files and modified with Inkscape (v1.0) to improve clarity and add annotation.

## Results

### First encounter of a TRDV-containing TRA chain

For a donor PBMC-derived T-cell culture that showed reactivity to a tumor cell line in an MLTC, we attempted to isolate the TR that conferred the observed reactivity. We were able to identify the TRBV using the Beta Mark kit (Beckman Coulter, Brea, CA, USA) (**Supplementary Figure S2**) and to amplify the nucleotide sequence of the TR beta chain using our standard amplification method with TRBV gene-specific forward and TRBC-specific reverse PCR primers (31). However, the TRA chain was refractory to this RT-PCR-based method using TRAV-specific forward primers. Therefore, we applied 5’RACE to amplify the TRA chain sequence (*cf*. *Materials and Methods* section, **Supplementary Table S2** for PCR primer sequences and **Supplementary Figure S2** for further experimental context). The amplification was successful, and Sanger sequencing of the 5’RACE product revealed a TR chain that encompassed 89 bp of Homsap TRDV1 5’-untranslated region (UTR), followed by the TRDV1 coding sequence that was joined to TRAJ24*03 by a 16-amino acid CALGDCITDSWGKFQF-encoding junction sequence from 2nd-CYS 104 (FR3-IMGT) to J-PHE 118 of the J-MOTIF F/WGXG (FR4-IMGT) (**Table 1**) (3, 15). The open reading frame from the TRDV1 start codon to TRAC codon 84.2 (Leu) in the DE turn (14) just upstream of the 5’RACE PCR primer binding site was intact, suggesting a productive TR.

**Table 1:** Composition of the TRDV-containing TR in this study.

Experiment	TR
Tumor-reactive human T-cell culture, 29.ct2	TRA chain: TRDV1 – CALGDCITDSWGKFQF – TRAJ24 – TRAC TRB chain: TRBV12-3 – CASSNSGGTIGGYNEQFF – TRBJ2-1 – TRBC2
Murine TILs, m25800.ct1	TRA chain: TRDV2-2 – CALMEPLNTGYQNFYF – TRAJ49 – TRAC TRB chain: TRBV12-2 – CASSGLGVIYEQYF – TRBJ2-7 – TRBC2

To analyze this MLTC in greater depth and identify additional clonotypes, we conducted 10X VDJ sequencing on archived T cells from an earlier time point of the same culture (day 14, **Supplementary Figure S2A**). The 10X CellRanger vdj pipeline identified the same TR beta chain amplified before by RT-PCR for the second most frequent clonotype of this T-cell culture (**Table 1**) but, seemingly, no TRA chain. Manually inspecting the files generated by the 10X CellRanger pipeline, we found no TRA chain in the final filtered results (vloupe file, filtered_contigs.csv, web_summary.html, etc.). However, the entries for this clonotype in the prefiltering “all_contig_annotations.csv” file showed TRDV1 joined to TRAJ24 as identified before by 5’RACE. Also, the junction sequence CALGDCITDSWGKFQF was identical to that obtained from the 5’RACE product. The cell barcodes unambiguously linked this TRA chain to the solitary TR beta chain of the final filtered 10X VDJ results. This TR will be referred to as 29.ct2 subsequently.

### Second encounter, a TRA chain containing Musmus TRDV2-2

In an independent experiment, we performed TR sequencing of TILs from a murine tumor model. The results indicated that the most frequent clonotype among the isolated TILs (clonotype 1) apparently contained no TRA chain. As with the human T-cell culture described above, we manually inspected the pre-QC “all_contig_annotations.csv” file generated by the 10X CellRanger vdj pipeline to search for a potential TRA chain that shared the cell barcodes of the apparently solitary beta chain. We found a TRA chain that shared cell barcodes with the clonotype 1 beta chain and joined Musmus TRDV2-2 to TRAJ49 via a 16-amino acid junction encoding CALMEPLNTGYQNFYF (or 14 amino acids CDR3-IMGT without C104 and F108; **Table 1**). We refer to this TR as m25800.ct1.

### TR chains possess intact CDS

Both TRA chains were categorized as nonproductive by CellRanger (‘productive=false’ in results files), suggesting a disrupted open reading frame that does not encode an intact protein. However, since the 5’RACE results indicated a TR chain with a functional coding sequence, we extracted the sequence assemblies from the respective “all_contig_annotations.json” files. We found that these sequences encode the TR V genes (including 5’UTR), CDR3 region, J gene, and 5’ part of the C domain [until Homsap TRAC codon 3 in the A strand and until Musmus TRAC codon 22 in the B strand, respectively (32)] all in-frame as an intact coding sequence. It can be inferred that the downstream part of the C gene is also intact [for technical reasons, the downstream parts of the C genes are not contained in 10X 5’ libraries used for TR sequencing, *cf.* reference (32)]. Therefore, we concluded that the TRDV-containing TRA chains contain complete intact open reading frames.

### TRs are expressed on the cell surface of T cells

The information in the “all_contig_annotations” files of both experiments enabled us to clone the TR cDNAs for functional testing. To this end, we used our previously established Golden Gate Assembly-based TR cloning system (23, 24) that inserts V-(D)-J genes upstream of murine C genes that contain an additional cysteine residue facilitating a disulfide bridge between the alpha and beta C domains. The amino acid sequences of both recombinant TRs are provided in **Supplementary Figure S3**.

**Figure 1:**
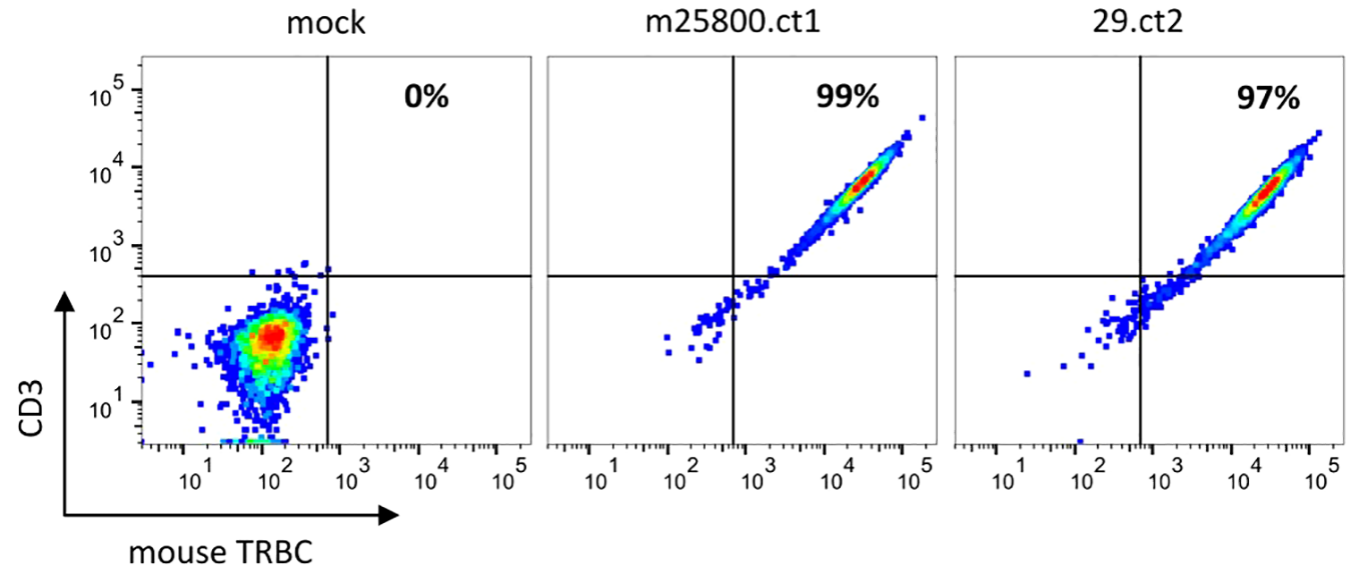
Flow cytometric analysis depicting the expression of recombinant TRs on JΔE10 Jurkat cells 48 h after transfection of *in vitro*-transcribed TR mRNAs. The percentages in the upper right quadrant enumerate those JΔE10 cells that are double-positive for TR and CD3. For each analysis, 10,000 cells were acquired.

To test the localization of the recombinant TR proteins to the cell surface, we used JΔE10 cells, Jurkat E6.1-derived cells in which both TR chains are disrupted by CRISPR/Cas9-introduced frameshift InDels in the respective C genes. The JΔE10 cells were transfected with *in vitro*-transcribed mRNA of the TR constructs and analyzed by FACS. As depicted in **Figure 1**, JΔE10 cells transfected with TR-encoding mRNAs show a strong signal for the murine TR C domain, whereas mock-transfected cells were not stained by this antibody. **Figure 1** also shows that mock-transfected JΔE10 cells are CD3-negative (left panel). Upon expression of the TR proteins, the cells become CD3-positive, indicating a colocalization of TR and CD3 at the cell surface.

### T cells transfected with TR 29.ct2 recognize tumor cells

**Figure 2:**
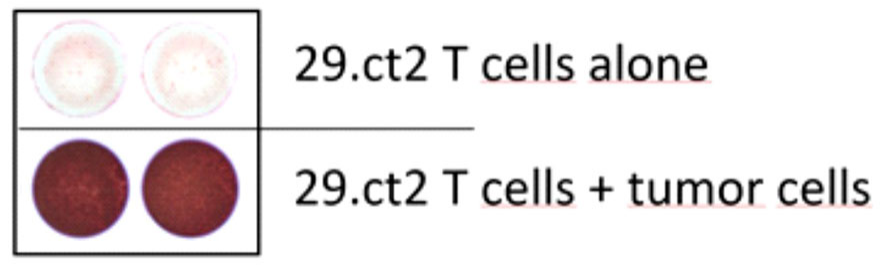
ELISpot assay of T cells transduced with T cell receptor 29.ct2. While the T cells show very limited IFNG secretion if incubated without tumor cells (top panel), coculture with tumor cells elicited a strong IFNG signal (bottom panel).

Despite the previously reported interaction of a TRDV-containing alpha-beta TR with a peptide-MH1 (pMH1) complex (12, 13) and despite the confirmation of productive rearrangements of the TR genes, we could not assume *ab initio* that the encoded TR proteins possessed TR signaling capability. Therefore, we introduced the 29.ct2 TR into primary T cells from a healthy blood donor after knocking out their endogenous TR chains (<2% residual expression, **Supplementary Figure S1**). The expression of the transgenic TR was verified by FACS analysis (**Supplementary Figure S1**). Subsequently, we used these T cells in an IFNγ ELISpot assay. Coculture of TR 29.ct2 transgenic T cells with the tumor cells resulted in a very strong release of IFNγ, while no relevant IFNγ production was observed without tumor cells (**Figure 2**). The recognition of the tumor cells by the TR 29ct.2 is HLA-independent, as the tumor cells were engineered to not express HLA molecules on their cell surface (*cf*. *Materials and Methods*).

The m25800.ct1 TR was found to be nonreactive in a FACS-based assay measuring CD107a on transgenic T cells after coculture with a cell line derived from the original mouse tumor.

Taken together, this demonstrates that the TRDV1-containing TR 29.ct2 confers binding of the T cells to the tumor cells and induces a very strong IFNγ response.

### TRDV-containing TRA chains are common in human and mouse

Next, we aimed to determine whether the two TRDV-containing TRs we discovered were rare events or whether TRDV genes are commonly incorporated into TRA chains. To this end, we queried the NCBI Sequence Read Archive (SRA) for 10X V-(D)-J data deposited in the form of Fastq files, downloaded and analyzed 43 randomly selected datasets, 21 of human and 22 of murine origin (**Supplementary Table S1**).

For example, the human V-(D)-J dataset SRR1311357 encompasses 12.38 million reads of which 8.04 million reads could be aligned to the Homsap TR loci. Those reads cover 104,950 TR cDNA molecules in 23,021 contigs. The CellRanger vdj analysis pipeline identified 5,961 clonotypes in this dataset (**Supplementary Table S3**, sheet 1) among which are clonotypes that comprise a TRDV-TRAJ TRA chain (**Supplementary Table S3**, sheet 2).

To quantify the subset of TRDV-containing TR clonotypes, we first extracted lines with the entry “TRDV” from the “all_contig_annotations.csv” file. Then, we filtered the entries according to the following criteria:

-  ≥2 UMIs (at least two TRDV-containing mRNAs in this cell have been reverse-transcribed and sequenced).
-  high_confidence = true (the CellRanger software assigned a high confidence to this contig).
-  full_length = true (the complete length of the targeted part of the open reading frame could be assembled from the sequencing reads).
-  is_cell = true (the software deemed this contig to originate from an actual T cell).
-  The “productive” criterion was disregarded as the two TRs we described above were deemed nonproductive by the CellRanger versions used (≤7.0.1).

Then, based on the cell barcodes of the TRDV-containing TRA chains, the other TR chain(s) of the respective cells were extracted and the resulting dataset was split into two fractions: (i) cells that exclusively contained a TRDV-TRA chain alongside a TR beta chain and (ii) cells with more than one TRA chain among which one contained a TRDV gene. For the first group, we manually copied the ‘raw_clonotype_id’ of the TR beta chain to the TRA chain with the same cell barcode because the TRDV-TRA contigs were not assigned the clonotype ID by the CellRanger software. The second group, harboring TRAV- as well as TRDV-containing contigs, was filtered, and all entries were deleted in which a “normal” TRAV-containing TRA contig had an equal or higher UMI count than the TRDV-containing contig. This left only those cells in which TRDV-TRA contigs were more abundant than their TRAV-TRA counterparts. The nonredundant TRAV-containing TRA repertoire of each dataset was used as a control set for statistical and abundance determinations.

We detected TRDV-containing TRA chains in 17/21 human and in 19/22 mouse repertoires. Those repertoires harboring clonotypes with hybrid TRA chains were on average more polyclonal (3,532 ± 3,070 clonotypes) than those in which we could not identify such TRA chains (188 ± 224 clonotypes) (**Supplementary Table S4**).

**Figure 3:**
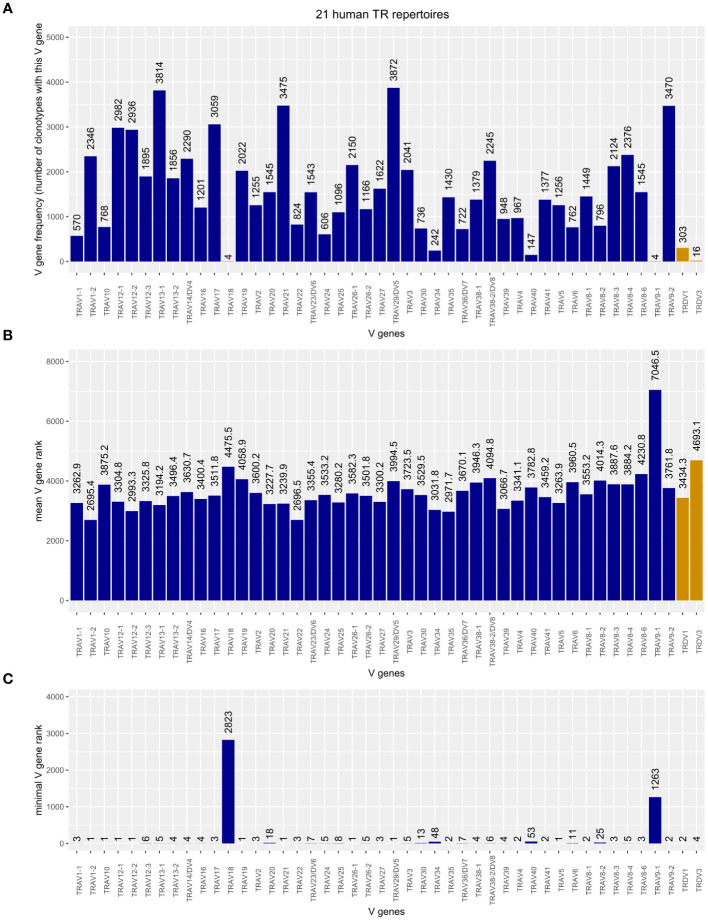
**(A)** Frequency plot of Homsap TRAV including TRAV/DV (dark blue) and TRDV (orange) genes in the TRA chains of 21 analyzed human TR repertoires. **(B)** Mean rank of clonotypes carrying the respective V gene. **(C)** Minimal, or “top,” rank of a clonotype carrying the respective V gene among the 21 human TR repertoires.

To obtain a comprehensively comparative picture of the TRDV usage in human TRA chains, we merged the two curated subsets described above and the TRAV-TRA repertoire of all 21 analyzed human datasets, retaining the sample ID for identification and correct clonotype rank/size calculation. This yielded 71,232 TRA clonotypes of which 349 (0.49%) contained TRDV genes. We found that TRDV1 and TRDV3 are incorporated into TRA chains; we did not identify a TRDV2-containing TRA chain that met our quality-filtering criteria. As shown in **Figure 3A**, TRDV1 is not very frequently used in V-J recombination of the TRA chain. However, it is more often used than the four TRAV genes (TRAV18, TRAV34, TRAV40, and TRAV9-1) while TRAV18 and TRAV9-1 were even more seldomly incorporated than TRDV3. When comparing clonotype sizes, for which we computed the mean rank of each V gene as a proxy, TRDV1 appears to be on par with the TRAV genes, whereas TRDV3 tends to be present in smaller clonotypes, only TRAV9‐1 being inferior in this respect (**Figure 3B**). **Figure 3C** depicts the minimal rank of each V gene rank, i.e., a V gene with rank = 1 represents the largest clonotype in the TR repertoire. The minimal V gene rank of TRDV1, rank = 2, refers to our in-house dataset “line29” where it is contained in the TRA chain of the second largest clonotype of the TR repertoire. Also, TRDV3 was found in a prevalent clonotype (rank = 4/fourth largest clonotype in the public dataset SRR13113846). This indicates that TRDV-containing clonotypes can become major clonotypes in TR repertoires.

**Figure 4:**
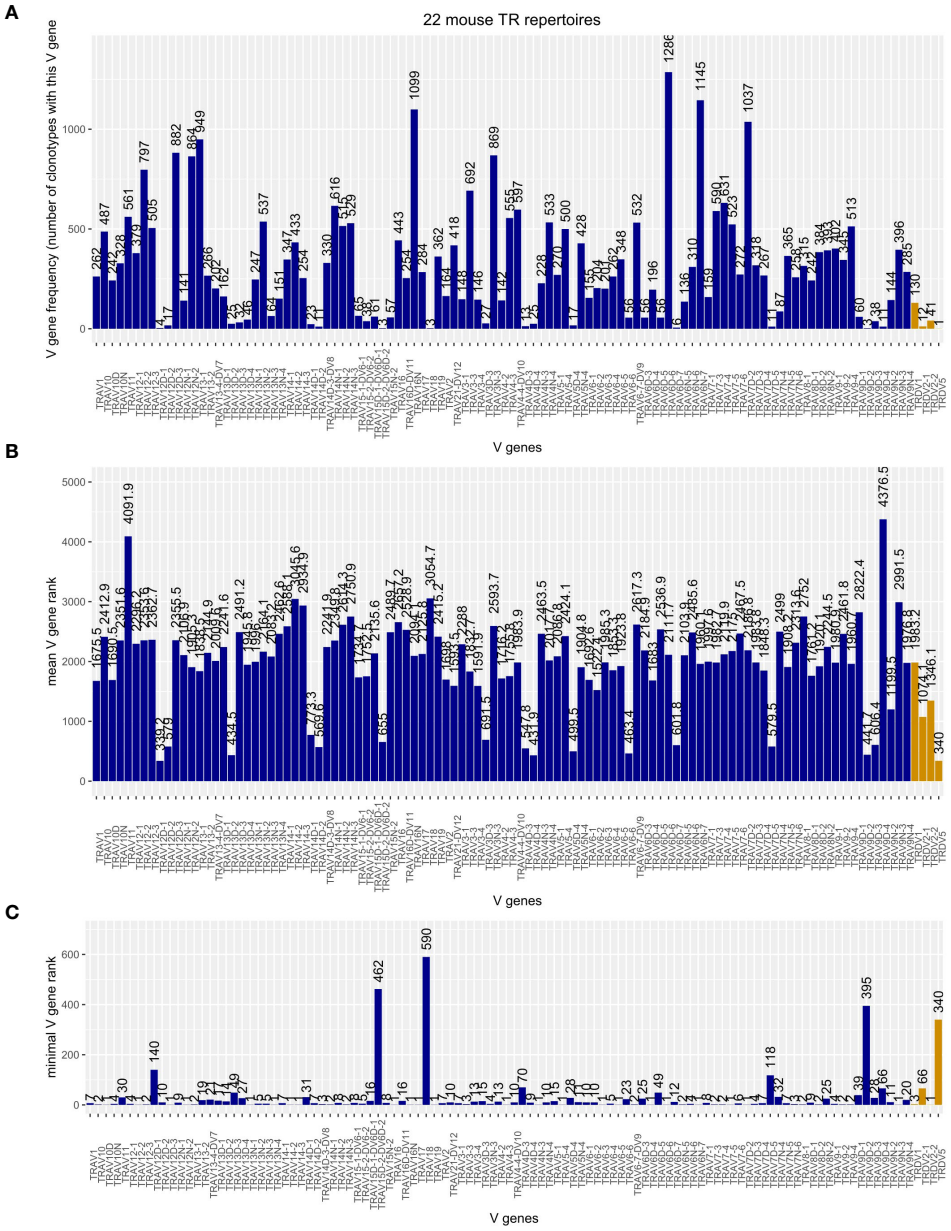
**(A)** Frequency plot of Musmus TRAV and TRDV genes in the TRA chains of 22 analyzed murine TR repertoires. **(B)** Mean rank of clonotypes carrying the respective V gene. **(C)** Minimal, or “top,” rank of a clonotype carrying the respective V gene among the 22 mouse TR repertoires.

Merging the TRA chains of the 22 mouse TR repertoires produced a table of 31,831 clonotypes, 184 (0.58%) of which carried a TRDV in their V domain. As shown in **Figure 4**, Musmus TRDV1, TRDV2-1, TRDV2-2, and TRDV5 are incorporated into murine TRA chains. Although there are 75 Musmus TRAV genes more abundant than TRDV1, the most often utilized TRDV gene, there are also 28 TRAV genes that are incorporated less frequently (**Figure 4A**). The frequency of utilization for TRDV2‐1 and TRDV2‐2 is in the same range as those of rarely incorporated TRAV genes like TRAV12D2‐2 and TRAV9D‐3, while TRDV5 was found in only one clonotype. If, however, a TRDV gene is incorporated into the TRA chain, the corresponding clonotypes are comparable in size to TRAV-containing TRA chains (**Figure 4B**) and can become large, shown as low minimal rank in **Figure 4C**: m25800.ct1, the largest clonotype in our TIL sample, includes TRDV2‐2; and two TRDV1-containing clonotypes are within the top 10 most abundant clonotypes of their respective repertoires (top rank = 3 in dataset SRR18687603, top rank = 5 in SRR18687609).

Musmus TRDV4 occurred in only one cell barcode in dataset SRR21989196. But in the same cell, a more abundant TRAV13D1-containing TRA chain was present (five and nine UMIs, respectively), so this chain did not meet our criteria. Nonetheless, there might be TR repertoires in which Musmus TRDV4 and, although we did not identify it in our screen, Musmus TRDV3 are productively incorporated into the TRA chains.

Taken together, our combined analyses of multiple human and mouse TR repertoires demonstrated that TRDV genes are incorporated into TRA chains at levels exceeding those of some TRAV genes. The sizes of the resulting clonotypes are on average comparable with those of TRAV-containing TRs and can become large, ranking in the top 10, or even dominate the repertoire as in our samples line 29 and m25800.

### J gene usage in TRDV-TRA and TRAV-TRA chains is similar

We were wondering whether there was a bias in the TRAJ utilization for TRAV-TRA and TRDV-TRA recombination events. As the number of individual TRDV gene-containing TRA chains is too small for a meaningful distribution analysis, we analyzed TRAJ frequencies of all identified TRDV-TRAJ vs. all identified TRAV-TRAJ TRA chains but found no statistically significant differences among human and mouse TRs (χ^2^ test p-value 0.25 in both cases). The TRAJ frequency plots are shown in **Supplementary Figure S4**. Interestingly, among the murine TRs, there appears to be a tendency to utilize more downstream-located TRAJ genes, independently of whether the V gene is a TRAV or a TRDV gene (**Supplementary Figure S4** bottom panel), whereas in the utilization frequency of human TRAJ genes, this trend seems to be overlaid several dips in frequency, e.g., between TRAJ24-TRAJ26, TRAJ32-TRAJ36, and TRAJ45-TRAJ48.

## Discussion and outlook

In this work, we identified two highly enriched TR clonotypes that contain TRDV genes in their TRA chains. Upon transfection into T cells, these TRs are translocated to the cell surface and the cells also become CD3-positive, suggesting the establishment of a TR-CD3 complex on the cell surface. While we could verify tumor cell line reactivity of the 29.ct2 TR by ELISpot, the m25800.ct1 TR was not reactive against an autologous tumor cell line. Explanations for the nonreactivity could be that the mutation evoking the expansion of the m25800.ct1 clonotype in the original tumor either is not contained in the tumor-derived cell line or was epigenetically silenced. Another possibility is that this T-cell clonotype was a nonreactive bystander that flourished in the tumor microenvironment for unknown reasons.

Despite the two TRs being highly expanded in their samples, representing the top 2/second largest (in Homsap) and top 1/largest TR clonotype, in Homsap and in Musmus, respectively, these could have been unique cases that, while interesting in themselves, bear no practical relevance for TR repertoire analysis. However, drawing from over 40 public high-throughput single-cell TR sequencing datasets, we showed that TRDV-containing TRA chains are present in the vast majority of TR repertoires. Actually, the datasets lacking this kind of TR ;chain tend to be smaller and more oligoclonal. This suggests that utilization of TRDV genes in V-(D)-J recombination to generate TRA chains is not an exception but rather common. Our analyses demonstrated that TRDV genes are not as frequently used as many TRAV genes, representing approximately 0.5% among human and 0.6% among murine TR clonotypes. However, especially Homsap TRDV1, Musmus TRDV1, and to some extent Musmus TRDV2-2 are more commonly incorporated into TRA chains than some TRAV genes, making the TRDV genes a relevant contribution to TRA diversity. Moreover, we observed TRDV-TRA chains among the top 10 clonotypes not only in our in-house datasets but also in public ones. This suggests that TRDV-containing clonotypes constitute a notable contribution to human and murine TR repertoires. Interestingly, while the absence of some TRDV genes from alpha-beta TRs could be explained by insufficient depth of our study, the absence specifically of Homsap TRDV2 from alpha-beta TRs might be due to a negative thymic selection during fetal and postnatal development (33).

The recombination of TRDV, or TRDD, with TRAJ genes does not violate the 12/23 rule (34) and has been proven experimentally to some extent (12, 13). However, utilization of V genes in TRA recombination is thought to be governed at least in part by features of their promoters and chromatin accessibility (35–38). While we found a tendency toward utilization of more downstream-located TRAJ genes, this was present in both TRAV-containing TRA chains and TRDV-TRA chains and we observed no apparent difference between the two types of alpha chains.

Our results give rise to new questions: Can, for example, gamma-delta T cells harbor TRAV genes in their TR delta chains? Another interesting aspect is the apparent co-occurrence of “normal” TRAV-TRA chains in the same cells that carry TRDV-TRA chains. The UMI counts of these cells suggest that both TR chains are expressed. Do those T cells display two distinct types of TR on their surface and are both functional? Or is the expression of one TRA chain regulated by posttranscriptional phenotypic allelic exclusion (39–41); and if so, which one?

We hope that the work presented in this article will further establish the incorporation of TRDV genes into productive human and murine TRs as a verifiable fact, thus facilitating work on such questions as well as generally broadening the field of TR repertoire analysis.

It may happen to our fellow colleagues working with TR repertoire data obtained from 10X VDJ sequencing that they encounter TR clonotypes that seem to contain only a TR beta chain with no accompanying alpha chain. For those cases, we humbly suggest to turn to the unfiltered “all_contig_annotations” files of the respective dataset. More often than not, these files harbor a TRDV-containing TRA chain sharing the cell barcode(s) of the apparently solitary beta chain seen in the filtered results. This TRDV-TRA chain may, together with the TR beta chain, encode a functional TR. Another work-around to identify TRDV-containing TRA chains from 10X Genomics VDJ data is described in **Supplementary Method 1**.

## Data availability statement

The data presented in the study are deposited in the NCBI Sequence Read Archive (SRA) repository, accession numbers SRR26068869 and SRR26070009.

## Ethics statement

For the tumor-reactive T cell culture, PBMCs from healthy donors were provided by the blood bank of our institution according to the guidelines approved by the local ethics committee (Ethics committee of the Medical Association of Rhineland-Palatinate, Mainz, Germany). All donors gave written informed consent.

## Author contributions

MV: Conceptualization, Data curation, Formal Analysis, Investigation, Methodology, Project administration, Resources, Supervision, Validation, Visualization, Writing – original draft, Writing – review & editing. EF: Data curation, Formal Analysis, Investigation, Methodology, Visualization, Writing – review & editing. SZ: Investigation, Resources, Writing – review & editing. RO: Investigation, Resources, Supervision, Writing – review & editing. AB: Data curation, Formal Analysis, Investigation, Writing – review & editing. JG: Investigation, Writing – review & editing. EH: Investigation, Writing – review & editing. TW: Investigation, Supervision, Validation, Writing – review & editing. CW: Data curation, Formal Analysis, Investigation, Methodology, Resources, Supervision, Validation, Writing – review & editing.
