# Supplementary material for: Identification of TRDV-TRAJ V domains in human and mouse T-cell receptor repertoires
Source: Front Immunol. 2023 Nov 23;14:1286688. doi: 10.3389/fimmu.2023.1286688 (PMC10702483; doi:10.3389/fimmu.2023.1286688)
Supplement: Supplementary file 1 [file DataSheet_1.pdf]

Supplementary Tables, Figures and Method

Table S1. 10X TR scRNA-seq datasets used in this study.

Run	# of Spots	# of Bases	Size	Published	TRDV?
human datasets					
SRR13113571	12,383,571	1.4G	439.5Mb	11.02.2021	yes
SRR13113572	12,343,428	1.4G	457.5Mb	11.02.2021	yes
SRR13113573	12,279,976	1.4G	451Mb	11.02.2021	yes
SRR13113574	12,522,200	1.5G	464.4Mb	11.02.2021	yes
SRR13113575	12,494,556	1.5G	442Mb	11.02.2021	yes
SRR13113576	12,445,697	1.5G	443.9Mb	11.02.2021	yes
SRR13113577	12,233,730	1.4G	433.3Mb	11.02.2021	yes
SRR13113578	12,254,598	1.4G	445.6Mb	11.02.2021	yes
SRR13113579	12,396,475	1.5G	451.9Mb	11.02.2021	yes
SRR13113580	12,240,464	1.4G	447.1Mb	11.02.2021	yes
SRR13113581	12,389,881	1.4G	454.4Mb	11.02.2021	yes
SRR13113582	12,362,087	1.4G	455.2Mb	11.02.2021	yes
SRR13113846	11,836,951	1.4G	427.9Mb	11.02.2021	yes
SRR15597465	112,194,993	33.7G	14.5Gb	28.08.2021	yes
SRR18810015	120,980,310	36.5G	11.5Gb	19.04.2022	no
SRR18810016	127,969,006	38.6G	12.2Gb	19.04.2022	no
SRR18810018	103,676,491	31.3G	9.6Gb	19.04.2022	yes
SRR20751884	7,825,549	907.8M	364.5Mb	12.09.2022	no
SRR20751886	3,044,169	353.1M	144.3Mb	12.09.2022	no
SRR20751888	5,436,674	630.7M	256.1Mb	12.09.2022	yes
SRR26068869 (line29)	7,039,540	957.4M	337.6Mb	09.11.2023	yes
murine datasets					
SRR10723081	19,700,199	6.1G	2.6Gb	31.12.2021	yes
SRR10723082	19,402,014	6G	2.5Gb	31.12.2021	yes
SRR10723083	18,654,691	5.7G	2.4Gb	31.12.2021	yes
SRR10723084	17,360,590	5.3G	2.2Gb	31.12.2021	yes
SRR14911004	53,488,826	16.5G	5Gb	12.01.2022	yes
SRR18687603	9,268,016	2.9G	1.2Gb	01.06.2022	yes
SRR18687605	13,877,267	4.3G	1.8Gb	01.06.2022	no
SRR18687606	24,680,878	7.6G	3.4Gb	01.06.2022	yes
SRR18687607	2,582,825	795.5M	317.9Mb	01.06.2022	yes
SRR18687608	6,799,908	2.1G	890.6Mb	01.06.2022	yes
SRR18687609	19,191,079	5.9G	2.4Gb	01.06.2022	yes
SRR18687610	14,406,809	4.4G	1.9Gb	01.06.2022	yes
SRR18687611	13,670,842	4.2G	1.8Gb	01.06.2022	yes
SRR18687613	1,318,371	406.1M	134.5Mb	01.06.2022	yes
SRR18687614	1,011,945	311.7M	103.3Mb	01.06.2022	yes
SRR18687616	4,880,677	1.5G	599.8Mb	01.06.2022	no
SRR18687617	4,391,533	1.4G	542.8Mb	01.06.2022	no
SRR18687618	2,249,013	692.7M	229.6Mb	01.06.2022	yes
SRR18687619	2,601,839	801.4M	323.4Mb	01.06.2022	yes
SRR21738977	4,808,794	1.5G	480.1Mb	10.10.2022	yes
SRR21989196	56,669,812	10.1G	3.1Gb	25.10.2022	yes
SRR26070009 (m25800)	171,171,067	19.9G	6.2Gb	09.11.2023	yes

Table S2. Primers used in this study.

Primer name	Primer sequence	Purpose
TSO	AAGCAGTGGTATCAACGCAGAGTACATrGrGrG *	template-switching oligo for reverse transcription
TSO_preampPCRpr	AAGCAGTGGTATCAACGCAGAGTA	adapter-specific primer, 1st round of 5'RACE PCR
TSO_nestedPCRpr	GCAGTGGTATCAACGCAGAGTACAT	adapter-specific primer, nested RACE PCR
hsTRAC-RT	TTCGGAACCCAATCACTG	reverse transcription of TR alpha chain
hsTRAC-R1	GCTTGACATCACAGGAACTTTCTGG	1st round of 5'RACE PCR
hsTRAC-R2	TGCTCTTGAAGTCCATAGACCTCATG	nested RACE PCR

* ‘r’ denotes ribonucleotides

Table S3. (external .xlsx file) The first sheet of this Excel file resembles the “all_contig_annotations.csv” file for sample SRR1311357. The second sheet contains annotation for selected clonotypes with hybrid TRDV-TRAJ TRA chains.

Table S4. Average number of clonotypes in repertoires with and without TRDV-TRAJ TR alpha chains. While hybrid TR chains have been identified in oligoclonal as well as polyclonal repertoires, the repertoire in which we did not find TRDV-TRAJ TR chains were more oligoclonal.

datasets	average number of clonotypes in TR repertoires <u>with</u> hybrid TRA chains	average number of clonotypes in TR repertoires <u>without</u> hybrid TRA chains
human	5366 ± 2847 (range 68-12427)	232 ± 297 (range 13-643)
murine	1891 ± 2259 (range 171-9740)	129 ± 92 (range 38-222)
human+murine	3532 ± 3070	188 ± 224

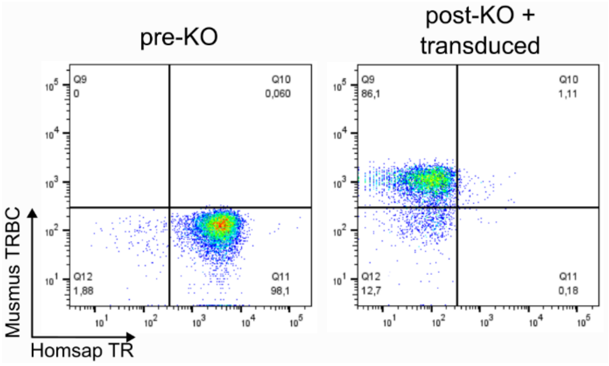

Figure S1. FACS analysis of endogenous TR knockout and transduction efficiency. Prior to CRISPR/Cas9-mediated knockout of the endogenous T cell receptor genes, the CD8$^+$ T cells among the donor-derived PBMCs stained 98% positive for TR (left plot). After knockout and retroviral transduction, only about 1% stain positive for Homsap TR (Q10+Q11 in the right plot) whereas 86% express the transgene-encoded T cell receptor (Q9 in right plot).

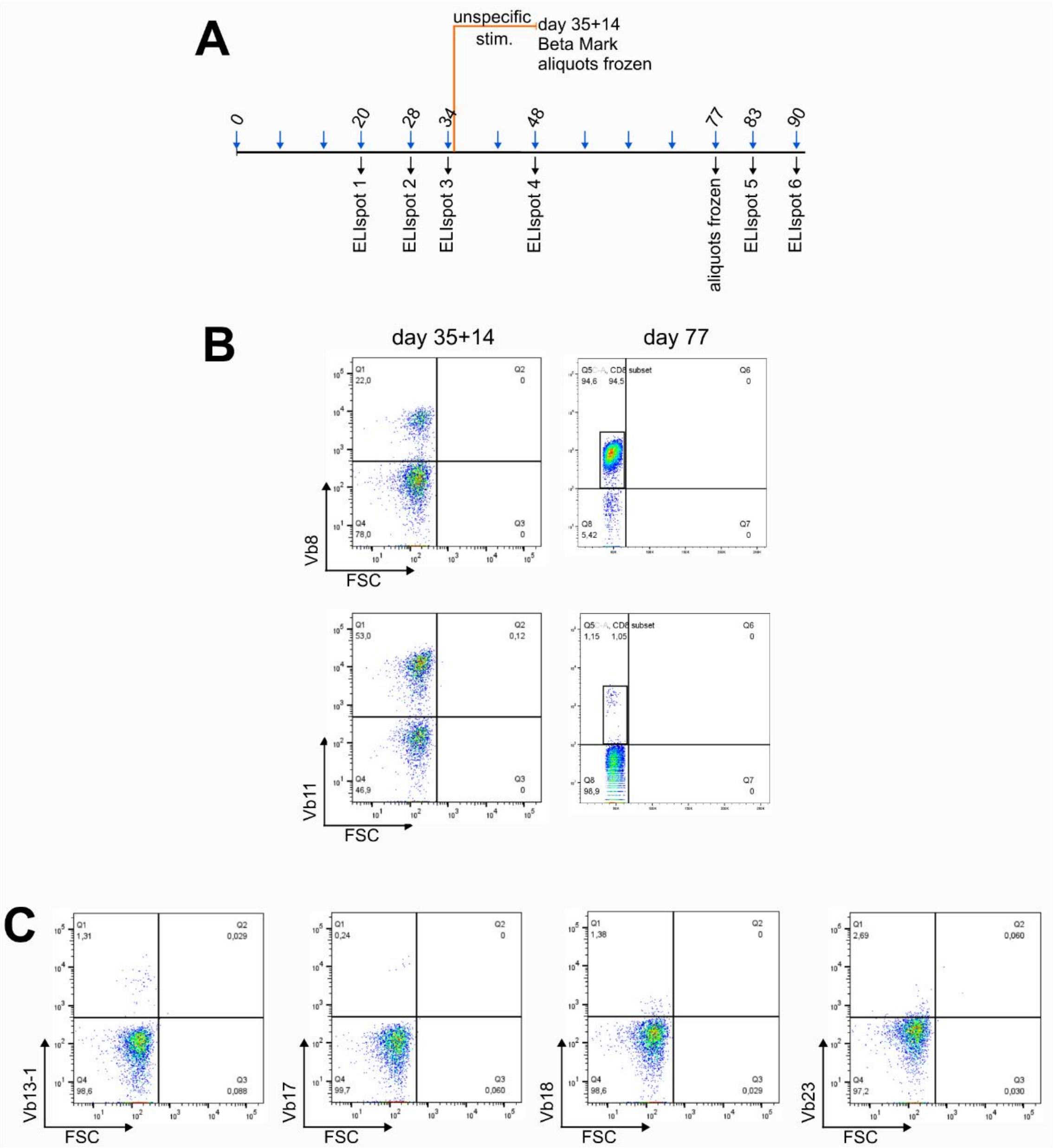

Figure S2. Mixed lymphocyte-tumor cell culture (MLTC) and Beta Mark^{TM} analyses. (**A**) Timecourse of the MLTC experiment. The blue arrows indicate the repeated stimulations with irradiated tumor cells. The black arrows mark aliquots taken from the culture for cryopreservation and ELIspot experiments. The orange line indicates a 14 days unspecific restimulation of the culture to expand it to a sufficient cell number for the Beta Mark FACS analysis and for cryopreservation. (**B**) Beta Mark results for antibody Vb8 (upper panel), detecting Homsap TRBV12-3, show an increase of this clonotype from 22% on day 35+14 to 94% on day 77. At the same time, Vb11 (lower panel), or Homsap TRBV25, which dominates the culture at day 35+14 (53%) decreases to 1% at day 77. (**C**) Additional Beta Mark results for other TRBV-containing TRB chains indicate the presence of a polyclonal population of TR clonotypes in the MLTC at this time. For each of the depicted scatterplots, data of 3300 cells was acquired.

Homsap TRDV1-containing TR   29.ct2

Homsap TRBV-(D)-J (TRBV12-3*01, TRBJ2-1*01 [5.6.16]), junction is underlined
Musmus TRB C-region (TRBC2*01, S79>C, C85.1)
P2A sequence
Homsap TRDV-TRAJ (TRDV1*01-TRAJ24*03 [7.3.14], junction is underlined, G strand 118-128 FGAGTQVVVTP
Musmus TRA C-region (TRAC*02, T84>C indicated by red background)
* above N residues signify potential N-linked glycosylation sites

peptide sequence:

```
MDSWTFCCVSLCILVAKHTDAGVIQSPRHEVTEMGQEVTLRCKPISGHNSLFWYRQTMMRGLELLIYFNNNVPIDDSGMPEDRFSAKMPNASFSTLKI
QPSEPRDSAVYFCASSNSGGTIGGYNEQFFGPGTRLTVLEDLRNVTPPKVSLFEPSKAEIANKQKATLVCLARGFFPDHVELSWWVNGKEVHSGVCTD
PQAYKESNYSYCLSSRLRVSATFWHNPRNHFRCQVQFHGLSEEDKWPEGSPKPVTQNISAEAWGRADCGITSASYQQGVLSATILYEILLGKATLYAV
LVSTLVVMAMVKRKNSGSGATNFSLLKQAGDVEENPGPGMLFSSLLCVFVAFSYSGSSVAQKVTQAQSSVSMPVRKAVTLNCLYETSWWSYYIFWYKQ
LPSKEMIFLIRQGSDEQNAKSGRYSVNFKKAAKSVALTISALQLEDSAKYFCALGDCITDSWGKFQFGAGTQVVVTPDIQNPEPAVYQLKDPRSQDST
                                                                                       *               *
LCLFTDFDSQINVPKTMESGTFITDKCVLDMKAMDSKSNGAIAWSNQTSFTCQDIFKETNATYPSSDVPCDATLTEKSFETDMNLNFQNLSVMGLRIL
LLKVAGFNLLMTLRLWSS*
```

nucleotide sequence of the CDS:

```
atggactcctggaccttctgctgtgtgtccctttgcatcctggtagcgaagcatacagatgctggagttatccagtcaccccgccatgaggtgacaga
gatgggacaagaagtgactctgagatgtaaaccaatttcaggccacaactccctttttctggtacagacagaccatgatgcggggactggagttgctca
tttactttaacaacaacgttccgatagatgattcagggatgcccgaggatcgattctcagctaagatgcctaatgcatcattctccactctgaagatc
cagccctcagaacccagggactcagctgtgtacttctgtgccagcagtaatagcgggggggacaatcggtggttacaatgagcagttcttcgggccagg
gacacggctcaccgtgctagaggatctgagaaatgtgactccacccaaggtctccttgtttgagccatcaaaagcagagattgcaaacaaacaaaagg
ctaccctcgtgtgcttggccaggggcttcttccctgaccacgtggagctgagctggtgggtgaatggcaaggaggtccacagtggggtctgcacggac
cctcaggcctacaaggagagcaattatagctactgcctgagcagccgcctgagggtctctgctaccttctggcacaatcctcgcaaccacttccgctg
ccaagtgcagttccatgggctttcagaggaggacaagtggccagagggctcacccaaacctgtcacacagaacatcagtgcagaggcctggggccgag
cagactgtgggattacctcagcatcctatcaacaaggggtcttgtctgccaccatcctctatgagatcctgctagggaaagccaccctgtatgctgtg
cttgtcagtacactggtggtgatggctatggtcaaaagaaagaactcaggaagcggagctactaacttcagcctgctgaagcaggctggtgacgtgga
ggagaaccctggacctggcatgctgttctccagcctgctgtgtgtatttgtggccttcagctactctggatcaagtgtggcccagaaggttactcaag
cccagtcatcagtatccatgccagtgaggaaagcagtcaccctgaactgcctgtatgaaacaagttggtggtcatattatatttttggtacaagcaa
cttcccagcaaagagatgattttccttattcgccagggttctgatgaacagaatgcaaaaagtggtcgctattctgtcaacttcaagaaagcagcgaa
atccgtcgccttaaccatttcagccttacagctagaagattcagcaaagtacttttgtgctcttggggactgcataactgacagctggggaaattcc
agtttggagcagggacccaggttgtggtcaccccagacatccagaacccagaacctgctgtgtaccagttaaaagatcctcggtctcaggacagcacc
ctctgcctgttcaccgactttgactcccaaatcaatgtgccgaaaaccatggaatctggaacgttcatcactgacaaatgcgtgctggacatgaaagc
tatggattccaagagcaatggggccattgcctggagcaaccagacaagcttcacctgccaagatatcttcaaagagaccaacgccacctaccccagtt
cagacgttccctgtgatgccacgttgactgagaaaagctttgaaacagatatgaacctaaactttcaaaacctgtcagttatgggactccgaatcctc
ctgctgaaagtagccggatttaacctgctcatgacgctgaggctgtggtccagttga
```

Musmus TRDV2-2-containing TR   m25800.ct1

Musmus TRBV-(D)-J (TRBV12-2*01, TRBJ2-7*01 [5.6.12], junction is underlined
Musmus TRB C-region (TRBC2*01, S79>C, C85.1)
P2A sequence
Musmus TRDV-TRAJ (TRDV2-2*01-TRAJ49 [7.3.14] junction is underlined, G strand 118-128 FGKGTSLTVIP
Musmus TRA C-region (TRAC*02, T84>C indicated by red background)
* above N residues signify potential N-linked glycosylation sites

peptide sequence:

```
MSNTAFPDPAWNTTLLSWVALFLLGTSSANSGVVQSPRYIIKGKGERSILKCIPISGHLSVAWYQQTQGQELKFFIQHYDKMERDKGNLPSRFSVQQF
DDYHSEMNMSALELEDSAVYFCASSGLGVIYEQYFGPGTRLTVLEDLRNVTPPKVSLFEPSKAEIANKQKATLVCLARGFFPDHVELSWWVNGKEVHS
GVCTDPQAYKESNYSYCLSSRLRVSATFWHNPRNHFRCQVQFHGLSEEDKWPEGSPKPVTQNISAEAWGRADCGITSASYQQGVLSATILYEILLGKA
TLYAVLVSTLVVMAMVKRKNSGSGATNFSLLKQAGDVEENPGPGMVRPFFLWVLFLSTSLEASMAQTVSQPQKKKSVQVAESATLDCTYDTSDTNYLL
FWYKQQGGQVTLVILQEAYKQYNATLNRFSVNFQKAAKSFSLEISDSQLGDAATYFCALMEPLNTGYQNFYFGKGTSLTVIPNIQNPEPAVYQLKDPR
                                                                                       *               *
SQDSTLCLFTDFDSQINVPKTMESGTFITDKCVLDMKAMDSKSNGAIAWSNQTSFTCQDIFKETNATYPSSDVPCDATLTEKSFETDMNLNFQNLSVM
GLRILLLKVAGFNLLMTLRLWSS*
```

nucleotide sequence of the CDS:

```
atgtctaacactgccttccctgaccccgcctggaacaccaccctgctatcttgggttgctctctttctcctgggaacaagttcagcaaattctggggt
tgtccagtctccaagatacataatcaaaggaaagggagaaaggtccattctaaaatgtattcccatctctggacatctctctgtggcctggtatcaac
```

agactcaggggcaggaactaaagttcttcattcagcattatgataaaatggagagagataaaggaaacctgcccagcagattctcagtccaacagttt
gatgactatcactctgagatgaacatgagtgccttggagctagaggactctgccgtgtacttctgtgccagctccggactggggttatttatgaaca
gtacttcggtcccggcaccaggctcacggttttagaggatctgagaaatgtgactccacccaaggtctccttgtttgagccatcaaaagcagagattg
caaacaaacaaaaggctaccctcgtgtgcttggccaggggcttcttccctgaccacgtggagctgagctggtgggtgaatggcaaggaggtccacagt
ggggtctgcacggaccctcaggcctacaaggagagcaattatagctactgcctgagcagccgcctgagggtctctgctaccttctggcacaatcctcg
caaccacttccgctgccaagtgcagttccatgggctttcagaggaggacaagtggccagagggctcacccaaacctgtcacacagaacatcagtgcag
aggcctggggccgagcagactgtgggattacctcagcatcctatcaacaaggggtcttgtctgccaccatcctctatgagatcctgctagggaaagcc
accctgtatgctgtgcttgtcagtacactggtggtgatggctatggtcaaaagaaagaactcaggaagcggagctactaacttcagcctgctgaagca
ggctggtgacgtggaggagaaccctggacctggcatggtacggccgttcttcctgtgggtgctcttcctttccacttctcttgaagccagcatggctc
agacagtgtctcagcctcagaagaaaaagtctgtgcaggtggcagaatcagcaaccctggactgcacctatgacacaagtgatactaattacctcttg
ttctggtacaaacagcaaggagggcaggtgactctcgtcattctccaagaagcatacaagcagtataatgcaacgttaaaccgcttctctgtgaactt
ccagaaagcagctaagtccttcagcctggagatctccgactcgcagctgggggatgctgcgacgtatttctgtgctctcatggagcctttgaacacgg
gttaccagaacttctattttgggaaaggaacaagtttgactgtcattccaaacatccagaacccagaacctgctgtgtaccagttaaaagatcctcgg
tctcaggacagcaccctctgcctgttcaccgactttgactcccaaatcaatgtgccgaaaaccatggaatctggaacgttcatcactgacaaatgcgt
gctggacatgaaagctatggattccaagagcaatggggccattgcctggagcaaccagacaagcttcacctgccaagatatcttcaaagagaccaacg
ccacctaccccagttcagacgttccctgtgatgccacgttgactgagaaaagctttgaaacagatatgaacctaaactttcaaaacctgtcagttatg
ggactccgaatcctcctgctgaaagtagccggatttaacctgctcatgacgctgaggctgtggtccagttga

Figure S3. Amino acid and nucleotide sequences of analyzed TRs. The nucleotide sequences contain silent mutations to remove unwanted restriction enzyme sites and/or alter codon usage. Sequences of the germline genes are provided in support of the TR V and C gene names, based on the IMGT unique numbering for V domain [1] and C domain [2], respectively. CDR-IMGT lengths of the rearranged genes in the V-beta domain and in the TRDV-TRAJ domain are shown between brackets separated with dots.

For the inclined reader, please use the IMGT Protein Display function (*e.g.* for Musmus TRAC https://www.imgt.org/IMGTrepertoire/Proteins/proteinDisplays.php?species=house%20mouse&latin=Mus%20musculus&group=TRAC) and IMGT DomainGapAlign (https://www.imgt.org/3Dstructure-DB/cgi/DomainGapAlign.cgi) for more extensive analyses and highlighting, *e.g.* of the introduced additional cysteine residue [3, 4], the introduced silent mutations or potential N-glycosylation sites.

A

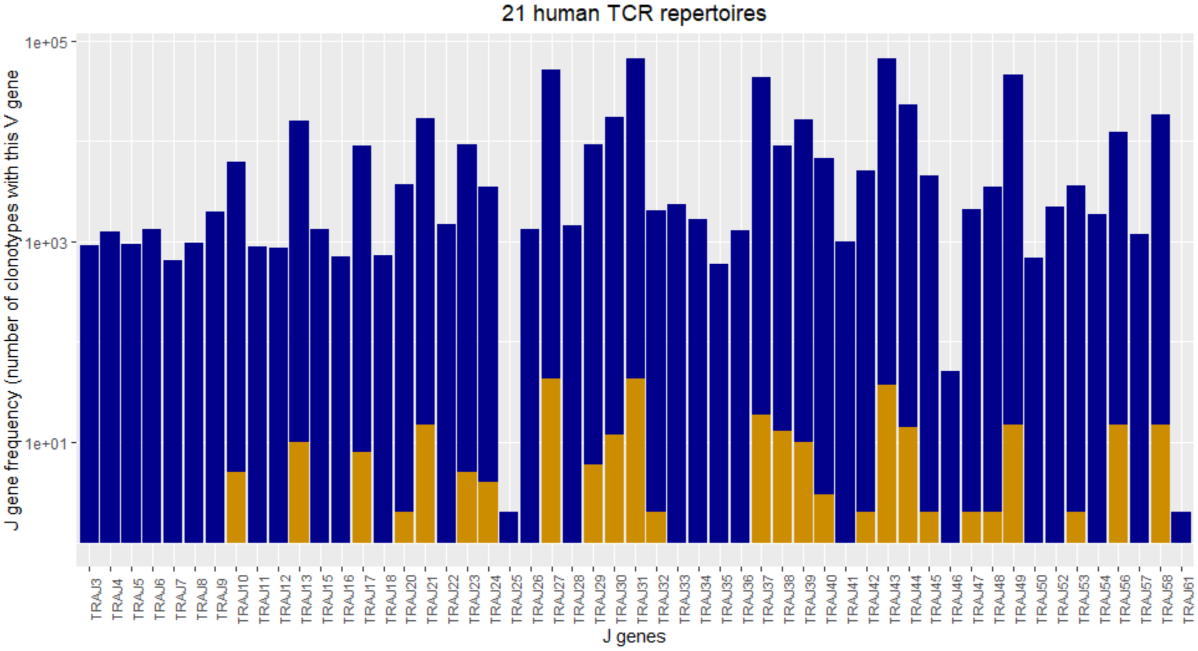

B

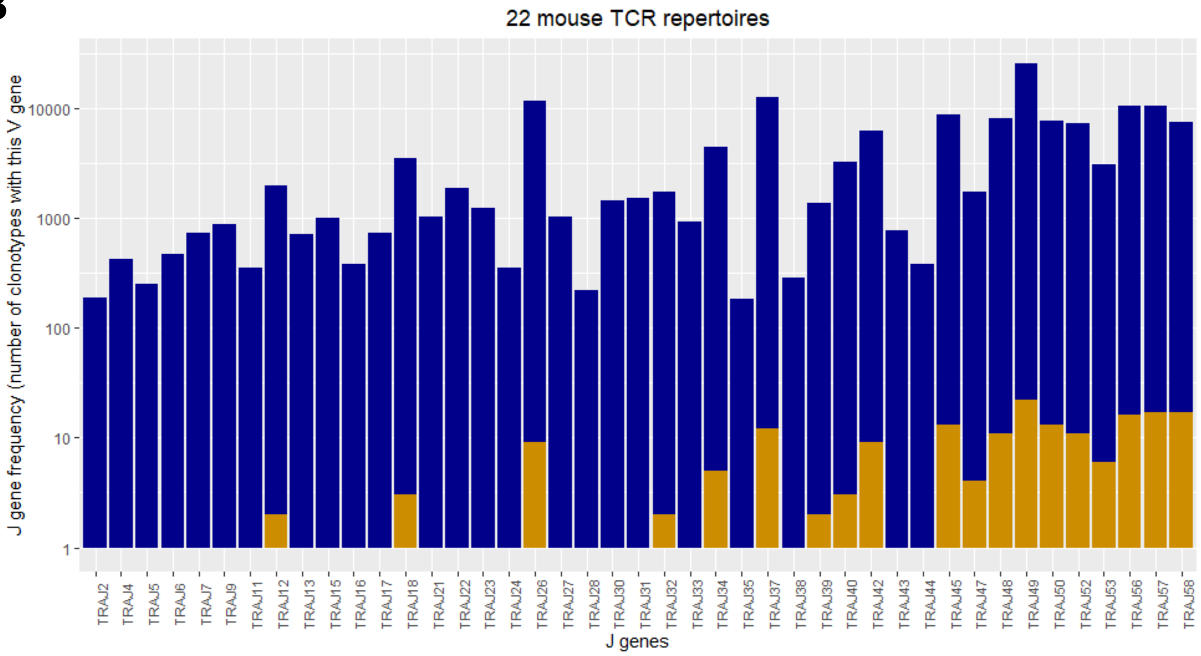

Figure S4. TRAJ frequency distribution among TRAV-containing (dark blue) and TRDV-containing (orange) TR alpha chains. Accumulated data of all human TR alpha repertoires is shown in (A), the murine repertoires are shown in (B). Note the log10 scale of the y axis.

Supplementary Method 1

While the manual identification of TRDV-containing TR alpha sequences in the unfiltered "all_contig" files output by CellRanger is formally appropriate it is also tedious and requires a lot of hands-on work. Hence, a more straightforward method might be desirable. The idea at the core of the method described below has been suggested by the 10X Research & Development team.

1. Download, or locate in your file system, the V-(D)-J reference Fasta file. For human, the file name reads as "`refdata-cellranger-vdj-GRCh38-alts-ensembl…`".
2. In the Fasta file, locate the TRDV… entries and rename them to TRAV… For example, rename TRDV1 to TRAV101, TRDV2 to TRAV102, … and save the edited Fasta file under a different name.
3. Use this alternative reference Fasta file in the CellRanger vdj pipeline if you observe seemingly solitary TR beta chains or otherwise suspect TRDV-containing TR alpha chains in your TR repertoire data.

In our hands, this workaround identified the hybrid TR alpha chains as productive.

Supplementary References

1. Lefranc, M.P., et al., *IMGT unique numbering for immunoglobulin and T cell receptor variable domains and Ig superfamily V-like domains.* Dev Comp Immunol, 2003. **27**(1): p. 55-77.
2. Lefranc, M.P., et al., *IMGT unique numbering for immunoglobulin and T cell receptor constant domains and Ig superfamily C-like domains.* Dev Comp Immunol, 2005. **29**(3): p. 185-203.
3. Cohen, C.J., et al., *Enhanced antitumor activity of T cells engineered to express T-cell receptors with a second disulfide bond.* Cancer Res, 2007. **67**(8): p. 3898-903.
4. Kuball, J., et al., *Facilitating matched pairing and expression of TCR chains introduced into human T cells.* Blood, 2007. **109**(6): p. 2331-8.
